# Use of the Perturbing Sphere Method for the Estimation of Radiofrequency Coils’ Efficiency in Magnetic Resonance Applications: Experience from an Electromagnetic Laboratory

**DOI:** 10.3390/s24175705

**Published:** 2024-09-02

**Authors:** Giulio Giovannetti, Francesca Frijia

**Affiliations:** 1 Institute of Clinical Physiology, National Council of Research, 56124 Pisa, Italy; 2 Bioengineering Unit, Fondazione Toscana G. Monasterio, 56124 Pisa, Italy; ffrijia@ftgm.it

**Keywords:** radiofrequency coils, magnetic resonance, workbench tests, coil efficiency

## Abstract

Radiofrequency (RF) transmitter and receiver coils are employed in in magnetic resonance (MR) applications to, respectively, excite the nuclei in the object to be imaged and to pick up the signals emitted by the nuclei with a high signal-to-noise ratio (SNR). The ability to obtain high-quality images and spectra in MR strongly depends on the RF coil’s efficiency. Local coil efficiency can be estimated with magnetic field mapping methods evaluated at a fixed point in space. Different methods have been described in the literature, divided into electromagnetic bench tests and MR techniques. In this paper, we review our experience in designing and testing RF coils for MR in our electromagnetic laboratory with the use of the perturbing sphere method, which permits coil efficiency and magnetic field mapping to be estimated with great accuracy and in a short space of time, which is useful for periodic coil quality control checks. The method’s accuracy has been verified with simulations and workbench tests performed on RF coils with different surfaces and of different volumes. Furthermore, all the precautions taken to improve the measurement sensitivity are also included in this review, in addition to the various applications of the method that have been described over the last twenty years of research in our electromagnetic laboratory.

## 1. Introduction

Magnetic resonance imaging (MRI) and magnetic resonance spectroscopy (MRS) are both non-invasive diagnostic techniques that exploit the nuclear magnetic resonance (NMR) phenomenon. MRI employs signals from tissues to produce anatomic images, while MRS uses this information to determine the concentration of metabolites in tissues [1].

The optimization of radiofrequency (RF) coils is a crucial aspect of magnetic resonance (MR) systems. To obtain high-quality MR imaging and spectroscopy data, RF coils must be able to generate a homogeneous magnetic field over a relatively large volume during transmission and to achieve a high signal-to-noise ratio (SNR) during reception [2]. According to their geometries, RF coils can be divided into volume coils, often used for both transmission and reception, and surface coils, mainly used as receivers [3]. The use of phased-array coils permits the achievement of a large region of sensitivity and a high SNR [4].

The efficiency of RF coils is an important indicator of coil performance and is defined as $B_{1}/\sqrt{P}$, where *B*_1_ is the RF magnetic field induced by the coil at a given point and *P* is the supplied power [5]. This parameter characterizes the performance of both transmitter and receiver coils in an empty coil due to the principle of reciprocity for MR in situations where no lossy sample is present [6]. It is important to note that maximizing the coil sensitivity will also maximize the SNR [7].

Both coil efficiency estimation and coil magnetic field mapping can be performed with probe techniques and imaging techniques. The former are able to measure field values at discrete points in space, which can be used for generating a *B*_1_ map, while the latter produce an image directly [8]. From a practical point of view, measurements performed with probe techniques consist of inserting a small probe into the oscillating magnetic field and by moving such a probe to specific points in space, typically on a predetermined grid. The measurement results represent the magnetic field at specific points in space. In particular, in this paper, we focus on the perturbing sphere method [9], which was initially applied to map the *B*_1_ fields of X-band microwave cavity resonators [10] and loop-gap resonators (LGRs) [11] for pulsed Electron Spin Resonance (ESR) spectroscopy applications. Next, the method was applied to map RF fields from LGRs for Continuous-Wave Electron Paramagnetic Resonance (CW-EPR) and from Alderman–Grant resonators for Proton–Electron Double-Resonance Imaging (PEDRI) applications [12]. He et al. [13] measured the *B*_1_ field strength at the center of a surface coil resonator designed for 3D EPR imaging applications using a 2 mm diameter metal sphere. Hirata et al. [14] applied the perturbing sphere method to measure the efficiency at the center of a 300 MHz circular loop resonator designed for CW-EPR spectroscopy and imaging in live rats, while Petryakov et al. [15] employed the method to characterize a 9.2 GHz resonator for X-band EPR spectroscopy. Microwave magnetic field mapping of a Uniform Field (UF) 34 GHz cylindrical TE01U cavity for EPR spectroscopy experiments was recently performed with a metallic sphere introduced into the cavity volume [16], while a workbench test was performed to evaluate the efficiency of an electronically tunable resonator operating at 750 MHz for CW-EPR imaging of a tumor-bearing mouse leg [17]. Very recently, a 0.15 mm radius brass sphere was applied to test X-band (9–10 GHz) microwave (MW) resonators for EPR spectroscopy applications [18]. In particular, the sphere was placed inside a small EPR tube which was moved by a stepper motor driven inside the resonance cavity to extract the *B*_1_ field distribution.

After a brief introduction to the perturbing sphere method theory and to the estimation of RF coil performance parameters at the workbench, in this work, we summarize our experience in designing and testing RF coils for MR applications in our radiofrequency lab with the use of the perturbing sphere method. The method was applied to different applications, such as comparing the performance of different coils and calculating magnetic field mapping. The method’s accuracy was also verified through simulations and workbench tests.

## 2. Perturbing Sphere Method

This method was designed to measure the electric and magnetic field distribution in a resonant electromagnetic cavity by evaluating the change in its resonant frequency after a perturbation of its boundaries. In particular, if a small metallic sphere is introduced into the cavity interior, it should perturb the frequency depending on the local electric and magnetic fields, and this perturbation can be used for measuring the field strength at the position of the perturbing sphere.

If *f*_0_ is the unperturbed cavity resonant frequency and *f*_1_ its perturbed frequency after the insertion of a sphere of radius *r*, this expression is valid [9]:

(1) $$4\pi r^{3}\left(E_{0}^{2}-\frac{1}{2}H_{0}^{2}\right)=\frac{f_{0}^{2}-f_{1}^{2}}{f_{0}^{2}}$$

where *E*_0_ and *H*_0_ are, respectively, the magnitude of the electric and magnetic fields in the perturbing sphere position inside the cavity. Equation (1) shows that the insertion of the sphere does not allow for the separate measurement of the electric and magnetic fields but only their combination. Therefore, it must only be used when we are interested in one type of field at a point where we know independently that the other type is zero. Otherwise, the use of small conductors with needle shapes is required for distinguishing the electric and magnetic field contributions [9].

From Equation (1), in a region of zero electric field *E*_0_, the magnetic field can be measured as:

(2) $$H_{0}=\sqrt{\frac{1}{2\pi r^{3}}\left(\frac{f_{1}^{2}-f_{0}^{2}}{f_{0}^{2}}\right)}$$

By introducing the flux density of the magnetic field, denoted by *B*, and remembering that *B* = *µ*_0_*H*, where *µ*_0_ is the permeability of free space (4π·10^−7^ H/m), Equation (2) can be written in terms of the rotating component of magnetic field *B*_1_ as [10]:

(3) $$B_{1}=\frac{1}{2}\sqrt{\left(\frac{\mu_{0}P}{\pi^{2}B_{-3dB}r^{3}}\right)\left(\frac{f_{1}^{2}-f_{0}^{2}}{f_{0}^{2}}\right)}$$

where *B_−_*_3*dB*_ is the full width at half-power of the reflected signal from the cavity (namely –3 dB bandwidth), and *P* is the power lost in the cavity, which is equal to the power incident on the cavity in perfectly matched conditions.

When employed for radiofrequency MR coil characterization, the perturbing sphere method consists of inserting a small metallic sphere into the coil cavity (for volume coils) or near the coil plane (for surface coils) and measuring the frequency shift *f*_1_ caused by the sphere perturbation with respect to the coil resonant frequency *f*_0_. Then, by inserting Equation (3) into the coil efficiency definition, it is possible to obtain the following equation:

(4) $$\eta =\frac{B_{1}}{\sqrt{P}}=\frac{1}{2}\sqrt{\left(\frac{\mu_{0}}{\pi^{2}B_{-3dB}r^{3}}\right)\left(\frac{f_{1}^{2}-f_{0}^{2}}{f_{0}^{2}}\right)}$$

Equation (4) is valid for linearly polarized coils, while the term 1/2 must be replaced with 1/$\sqrt{2}$ for circularly polarized coils because the power will be split to drive the two quadrature channels [19]. It is important to remember that, based on the previous considerations, Equation (4) is valid only when the sphere is placed in a zero-electric field region. Moreover, since the MR sensitivity depends only on the transverse (x- and y-oriented) components (e.g., along the center axes of the empty birdcage and circular loop coils), the longitudinal (z-oriented) component of the *H* field must be zero.

## 3. Workbench Measurements

The quality factor, employed for providing a quantitative measure of coil quality, is defined as [20]:

(5) $$Q=2\pi \frac{energy\;stored\;in\;i-th\;cycle}{energy\;dissipated\;during\;i-th\;cycle}$$

Which can be calculated as:

(6) $$Q=\frac{2\pi f_{o}L}{R}$$

where *f*_0_ is the coil resonant frequency, *L* is its inductance, and *R* represents the coil losses.

From a practical point of view, the coil *Q* factor can be measured as:

(7) $$Q=\frac{f_{0}}{B_{-3dB}}$$

where *B_−_*_3*dB*_ is the –3 dB coil bandwidth.

**Figure 1:**
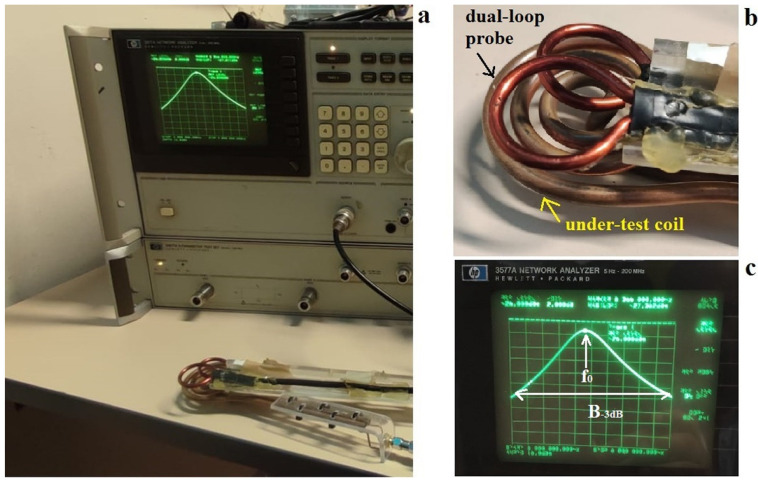
Coil workbench test: (**a**) measurement apparatus overview; (**b**) dual-loop probe placed on the under-test coil; (**c**) under-test coil response frequency.

The coil resonant frequency and –3 dB bandwidth can be easily measured using a network analyzer and a dual-loop probe constituted by two pickup loops (see Figure 1).

Such a setup is characterized by the absence of direct contact between the coil and the measurement instrument. The two circular loops that constitute the dual-loop probe are partially overlapped to minimize their mutual coupling: the loop centers are fixed at a distance of about 0.75 times their diameter [21]. The measurements on the under-test coil are performed by using one loop as a transmitter and the other as a receiver: the transmitter loop is weakly coupled to the under-test coil, which in turn is weakly coupled to the receiver loop. The power transmitted to the receiver loop is proportional to the oscillation amplitude of the under-test coil and, therefore, represents its frequency response [5].

**Figure 2:**
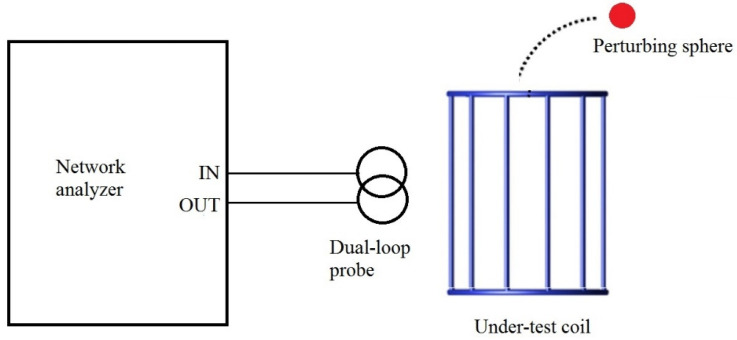
Experimental set up for coil efficiency measurements with perturbing sphere method.

Efficiency measurements with the perturbing sphere method (Figure 2) must be performed with the coil input ports open, and Equation (4) is valid when the coil and transmission line are perfectly matched. The measured *B_−_*_3*dB*_ bandwidth has to be multiplied by a factor of two before inserting its value into Equation (4). Moreover, to improve measurement accuracy, the network analyzer must be employed with a small frequency span and set in averaging mode. Hence, each efficiency value has to be calculated as the average of several measurements. Finally, it is necessary to ensure that the sphere support is made of a low-dielectric, nonconducting material so that it does not disturb the magnetic field. Regarding the choice of the sphere radius, the frequency shift increases with the sphere radius. Therefore, the variations in the coil resonant frequency are easier to detect when the sphere size increases. However, a larger sphere is associated with a larger measurement volume and a lower spatial resolution. Conversely, a small sphere should be used to obtain accurate magnetic field mapping. However, the frequency shift amplitude decreases and might be difficult to measure accurately, making the results more sensitive to the measurement noise [22]. In all experimental measurements performed in our laboratory and described in the following section, a compromise in the sphere size was found by considering the previously described constraints.

## 4. Coil Efficiency Measurements Performed in Our Lab

RF coils for MRI employ copper conductors with two different cross-sectional geometries, i.e., a circular wire and a flat strip (hereafter named “wire” and “strip”, respectively). The current distribution in the strip is less uniform than in the wire due to the lateral skin effect [23], and the strip thickness should be at least six times the skin depth to maximize the surface where the current flows, minimizing the conductor resistance [24].

Additionally, to maximize coil performance, the capacitors for coil tuning and matching are specially designed to be nonmagnetic and have high-quality characteristics [25].

Giovannetti et al. [26] compared four lowpass birdcage coils with identical dimensions (11 cm height and 14 cm diameter) and resonant frequencies (7.66 MHz). The first birdcage employed commercially available ceramic low-quality capacitors and was built using a 1 cm width and 35 µm thickness strip conductor. The second coil employed the same conductor but was tuned with high-quality capacitors (Qc > 10.000 at 1 MHz) developed by ATC. The third birdcage was built with high-quality capacitors and a 1 cm width strip conductor but with a thickness (800 µm) much higher than the penetration depth at the working frequency (23 µm). The fourth coil employed high-quality capacitors and was built with a 4.5 mm diameter wire conductor, providing the identical inductance of the strip.

As summarized in Table 1, workbench tests performed with the different birdcages showed that the coil employing standard-quality capacitors provided a very poor performance (Q = 21 and η = 11.92 µT/W^1/2^), while the performance greatly improved using the high-quality capacitors supplied by ATC (Q = 228 and η = 34.61 µT/W^1/2^).

The performance improved further when using strips with a thickness much higher than the penetration depth (Q = 374 and η = 42.74 µT/W^1/2^). However, the best performance was obtained using the wire conductor (Q = 477 and η = 52.31 µT/W^1/2^), thanks to the decrease in conductor resistance.

The workbench tests were performed using a metallic perturbing sphere of 6 mm radius. The accuracy of the perturbing sphere method was verified by using the ratio between η and √Q. In particular, the choice of conductor sizes [2] ensured that the reactive energy stored in the coils was the same. Both the Q factor (Equation (6)) and efficiency η (Equation (4), where P is proportional to the R conductor resistance [27]) indicate a variation in coil resistance. Therefore, the ratio between η and √Q should be almost constant, as shown in Table 1.

The perturbing sphere method was successively applied for testing two different ^13^C quadrature birdcage coils tuned at 32.13 MHz and designed for hyperpolarized MR studies at 3T [28]. The smaller coil (4 cm in radius and 12 cm in length) was employed for ^13^C metabolic studies in rats, while the bigger one (18.2 cm in radius and 36 cm in length) was used for pig experiments. Both sensitivities were evaluated with the coils in loaded conditions (2 g/L NaCl and de-ionized water 20 L cylindrical phantom for the pig coil and 9 g/L NaCl and de-ionized water 45 mL cylindrical phantom for the rat coil).

In order to verify the influence of the conductive material used for the sphere on the efficiency measurements, each coil was tested with two different sphere materials: the rat coil was tested with two 6 mm radius spheres, constituted by, respectively, steel and lead, while the pig coil was tested using a 20 mm radius steel sphere and a 17 mm radius lead sphere. Moreover, the same lead spheres were employed to perform efficiency measurements when coils were placed inside the MR scanner. The calculated efficiency values are summarized in Table 2.

The slight discrepancy between the measurements obtained with lead and steel spheres (below 3.2%) was associated with differences in sphere resistive losses.

The accuracy of the measurements was evaluated by taking into account the coil noise behavior. By assuming sample loss dominance for the loaded coil, the noise increases as the square root of the coil volume [29]. As the supplied power in Equation (4) depends on the noise resistance [27], the ratio between the square root of the pig and rat coil volumes (7.9) was seen to be in reasonable agreement with the ratio between the measured rat and pig coil efficiencies made on the bench (see Table 2, first two lines).

After the workbench tests, the two birdcages were employed for the acquisition of spectroscopic data on a cylindrical phantom containing 10 g [1-^13^C] acetate, 58 mL H_2_O, and 0.5 mmol Dotarem inserted in the center of the coils with its axis parallel to the coil axis. The SNR values of the [1-^13^C] acetate spectra were 1192 for the rat coil and 72 for the pig coil. The ratio between the pig coil spectra SNR and the rat coil spectra SNR was 16.5, in good agreement with the efficiencies η ratio evaluated inside the scanner (see Table 2). The comparison between the rat/pig coil η ratio measured inside and outside the scanner underlined the increase in pig coil losses inside the magnet, which was confirmed by the SNR. The authors concluded that this may depend on the position of the pig coil in the magnet, which was actually close to the top of the MR scanner body coil, producing a coupling between the two coils.

A comparison between two 7.5 cm radius circular coils (one built with 0.45 cm width and 40 µm thickness strip and the other with 0.1 cm radius wire) with identical inductance values and tuned to 42.6 MHz was performed by using a metallic perturbing sphere of 11.5 mm radius [30].

Table 3 shows the workbench test results.

The wire coil’s efficiency was 28% greater than the strip coil’s, which confirmed that the performance of this type of conductor was better. As previously described, the accuracy of the perturbing sphere method was verified with the equivalence of the ratio between η and Q^1/2^ for the two tested circular coils.

**Figure 3:**
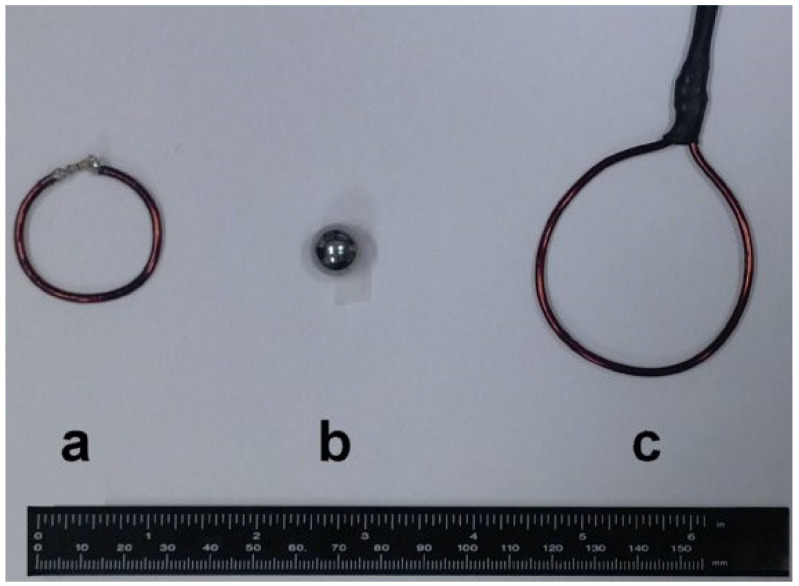
The three probes used for the coil efficiency measurements: (**a**) perturbing loop; (**b**) perturbing sphere; (**c**) pickup coil. Reprinted from [31].

A comparison between four coil efficiency estimation methods was described in [31]. In particular, three of the methods were based on efficiency measurements with “probe techniques” (perturbing loop, perturbing sphere, and pickup coil), which were able to generate *B*_1_ maps from different points in the space (see Figure 3), while the fourth (nutation experiment) was classified as an NMR technique.

The perturbing loop method provides additional loading via an inductive loop of copper wire in series with a known resistance placed inside the coil [32]. Compared with the perturbing sphere method, which requires an MR-compatible conductive material sphere for performing measurements inside the scanner, such a measurement technique can be used both on the bench and inside the scanner because it uses no magnetic parts. Moreover, due to the absence of working frequency limitation and without constraints in the coil geometry, the perturbing loop method can be employed for measurements in coils tuned to any magnetic field strength.

The pickup coil method measures the magnetic field in a coil by detecting the induced current [7], and it has been applied to imaging coils employed in MRI [27]. Such a technique can also be used for mapping the magnetic field in a nonresonant coil before inserting the tuning circuit [8].

A nutation experiment, considered the standard way to calibrate the RF magnetic field in MR, assesses the RF pulse width when the flip angle equals 360° and permits B_1_ estimation [33]. By calculating the power needed for a given spin nutation with a given excitation pulse, such a method provides the exact B_1_ values, but it is rather tedious. All approaches were tested on a quadrature lowpass birdcage coil (4 cm in radius and 12 cm in length) tuned to 32.13 MHz for ^13^C metabolic studies in rats. The coil efficiency was evaluated with the birdcage in the loaded condition (a cylindrical phantom with dimensions 2.5 cm diameter and 10 cm length containing 10g [1–^13^C] acetate, 58 mL H_2_O and 0.5 mmol Dotarem) and by placing the coil on a 3 T HDx TWINSPEE (GE Healthcare, Waukesha, WI, USA) scanner.

Table 4 summarizes the efficiency values measured with the four methods. The measurement with the perturbing sphere was performed with a 6 mm radius lead sphere and by calculating the efficiency value as the average of five measurements. The results for both the perturbing sphere and the perturbing loop method were reported as the mean value ± standard deviation.

The good agreement between the four efficiency estimation methods is evident because the differences between the measured values were below 5.2%. Regarding the perturbing sphere method, the experiment underlined that measurements are accurate only if the electric and the magnetic field components are well separated in space or in regions with a zero electric field value (i.e., for low-frequency tuned coils).

**Figure 4:**
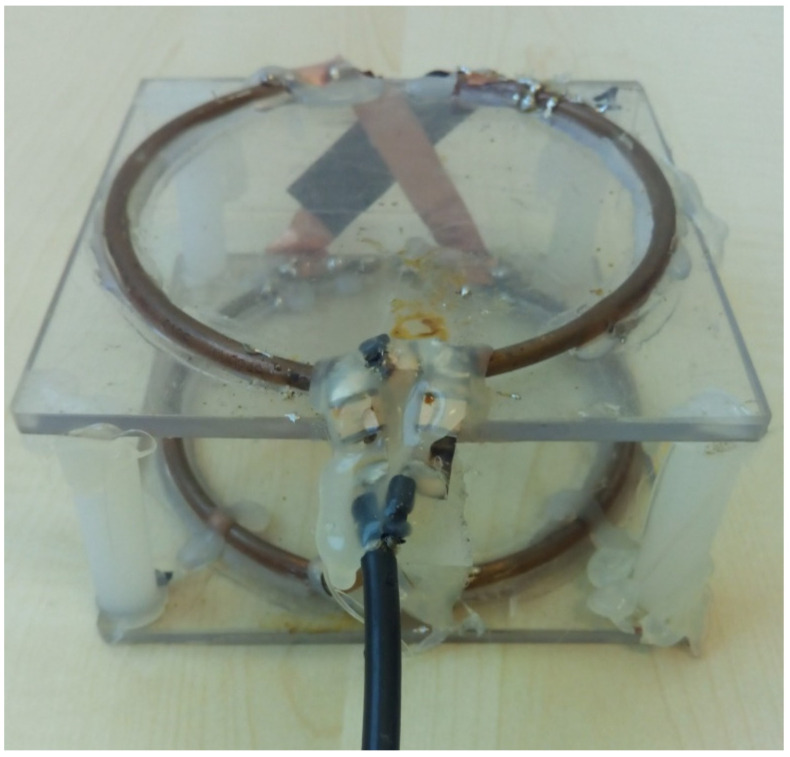
The Helmholtz coil prototype.

More recently, the efficiency of a prototype of a ^1^H transmit/receive 5.25 cm radius Helmholtz coil (Figure 4), suitable for MRI/MRS studies in small phantoms with a clinical 3T MR scanner, was tested by using a metallic perturbing sphere of 6 mm radius, resulting in a value of 4.82 μT/W^1/2^ in the coil’s center [34].

In [35], the simulation and design of a ^1^H transmitter/receiver with a 4 cm diameter circular coil for MRS studies in small phantom and animal models with a 3T MR scanner were described. Workbench tests performed on the home-built prototype, tuned at 127.75 MHz, included an efficiency estimation using the perturbing sphere method. With the electric field being negligible in the coil axial direction (z-axis), the magnetic field mapping was estimated by varying the sphere (6 mm radius) position, with the efficiency values being calculated at two z-coordinates along the coil’s axis.

**Figure 5:**
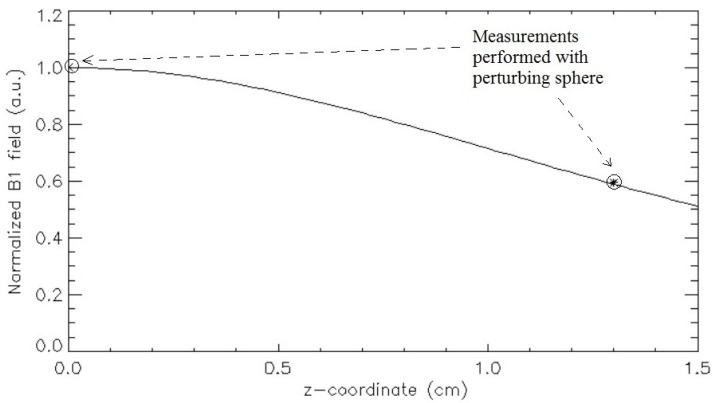
Circular coil simulated normalized magnetic field in the z-axis direction with the indication of the coil normalized efficiency values measured with the perturbing sphere method.

Table 5 shows the measured efficiency values as the mean value ± standard deviation. Figure 5 depicts the simulated normalized magnetic field in the z-axis and the normalized efficiency values measured with the perturbing sphere method.

The equivalence between the ratio of the coil efficiency values obtained at the two different z-coordinates (efficiency at z = 0 cm/efficiency at z = 1.3 cm, resulting equal to 1.68) and the ratio between the coil’s magnetic fields (Figure 5) calculated at the same z-coordinates (field at z = 0 cm/field at z = 1.3 cm, resulting equal to 1.69) underlined the accuracy of the perturbing sphere method. As depicted in Figure 5, the perturbing sphere method was described as able to provide, in a fast and easy way, RF coil field mapping. Moreover, it can also be useful for carrying out periodic coil quality control in a short amount time.

**Figure 6:**
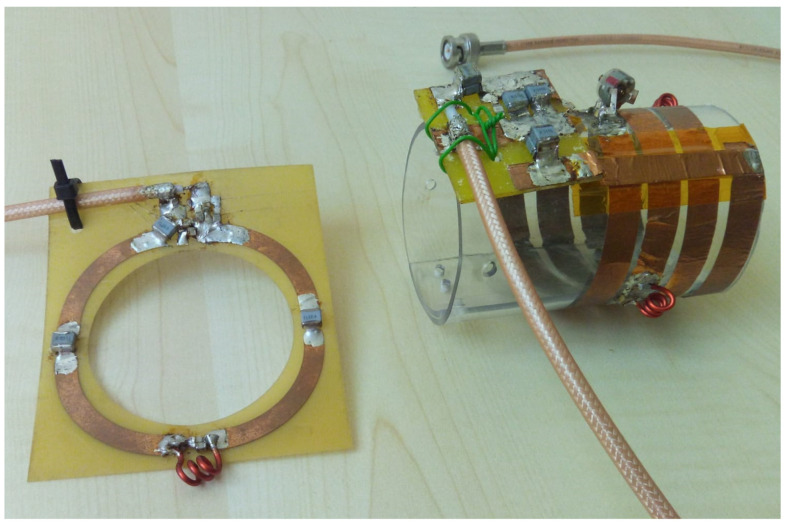
The ^13^C coil prototypes: on the left side, a circular loop; on the right side, the solenoid coil.

Frijia et al. [36] recently provided a detailed characterization and a comparison of a 3.5 cm radius circular coil and a 2.75 cm radius/4.5 cm length/four turns solenoid, both designed to be integrated with a clinical 3T MR scanner for hyperpolarized ^13^C studies in small animal models (Figure 6).

The workbench tests comprised coil efficiency evaluation using the perturbing sphere method, using a 3 mm radius metallic sphere placed at the center of the circular loop and the solenoid. For such measurements, the network analyzer was set to the averaging mode (32 averages) in order to improve the measurements’ sensitivity, and the coil efficiency values were calculated as the average of four measurements and reported as the mean ± standard deviation of such measurements, resulting in 72.05 ± 1.47 and 30.11 ± 0.76 μT/W^1/2^ for, respectively, the ^13^C solenoid and the ^13^C circular loop.

**Figure 7:**
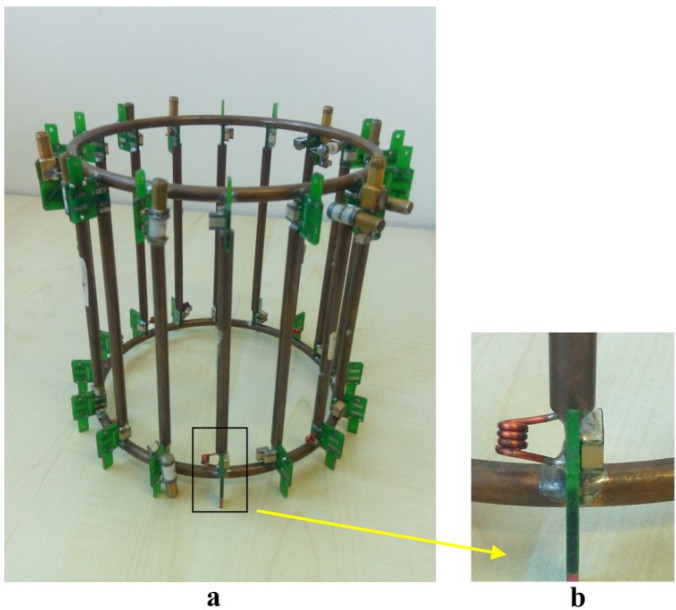
The ^1^H/^23^Na dual-tuned birdcage: (**a**) prototype overview; (**b**) trap circuit for channels decoupling.

In a recent work [37], the authors proposed a hardware and software setup for potentially evaluating ^23^Na MRI with a 3T clinical scanner. The employed coil was a dual-tuned ^1^H/^23^Na lowpass birdcage coil (15 cm length, 15 cm diameter) with alternate tuning of the legs [38] and trap circuits for decoupling the H and Na channels [39] (Figure 7).

The birdcage coil’s efficiency was measured using an 11.5 mm radius steel sphere for ^1^H and ^23^Na frequencies. The values, calculated as the average of four measurements with a standard deviation, resulted in 12.76 ± 0.99 and 1.30 ± 0.06 μT/W^1/2^ at frequencies of 33.78 MHz (^23^Na) and 127.75 MHz (^1^H), respectively.

Such a difference in the performances at the two frequencies was explained by the lower conductor resistance and higher capacitor quality factor provided at the ^23^Na frequency and the losses in the traps at the ^1^H frequency.

## 5. Discussion

Starting from a brief overview of the electromagnetic theory, in this paper, we summarize the experience of our electromagnetic laboratory with the perturbing sphere method, which is useful for estimating RF coil efficiency, and whose value strongly affects the SNR in MRI and MRS experiments. Since the measurements represent the field at specific points in space, the perturbing sphere method allows the RF coil’s magnetic field to be mapped in the form of a contour map or an image of the *B*_1_ field. Clearly, the spatial resolution of the technique is limited by the sphere size, the spatial density of the samples, and the positioning device’s accuracy. The sphere can be made of any conductive material (gold, silver, steel, or lead, with the last two being employed in the works described in this paper). However, when performing measurements inside an MR scanner, the use of an MR-compatible conductive material sphere is required. In this case, by comparing coil efficiency values measured inside and outside the MR scanner, it is also possible to evaluate any increase in losses when the coil is placed inside the magnet due to coupling with the scanner body coil and, therefore, justify the signal loss that occurs in MR acquisition compared to the expected value. The positioning device supporting the sphere must be made of a low-dielectric, nonconducting material (typically glass or Teflon). Furthermore, while useful for quick characterizations and comparisons, bench measurements of the efficiency of transmission and the sensitivity of receiving in an empty coil can give very different results than the sensitivities of the coil when loaded with a lossy sample [40].

Finally, because the perturbing sphere method is sensitive to both electrical and magnetic field components, it can only accurately estimate coil efficiency and magnetic field mapping if the electrical and magnetic field components are well separated in space.

We believe this paper contains information that is useful for designing and testing MR coils and, in addition, could also be useful for clarifying the importance of using an accurate method for both characterizing coil performance in workbench tests and for carrying out effective periodic coil quality control tests [41,42].

## Figures and Tables

**Table 1:** Workbench test results for the four birdcages [26].

Birdcage Typology	Q	η (μT/W^1/2^)	$\eta /\sqrt{Q}$
35 μm thickness strip conductor and standard-quality capacitors	21	11.92	2.6
35 μm thickness strip conductor and high-quality capacitors	228	34.61	2.2
800 μm thickness strip conductor and high-quality capacitors	374	42.74	2.3
Wire conductor and high-quality capacitors	477	52.31	2.4

**Table 2:** Workbench test results for the rat and pig coils [28].

Experiment	Rat Coil η (μT/W^1/2^)	Pig Coil η (μT/W^1/2^)	Rat/Pig η Ratio
Workbench efficiency test with steel sphere	20.92	2.21	9.47
Workbench efficiency test with lead sphere	20.66	2.28	9.06
MR scanner efficiency test with lead sphere	21.89	1.48	14.8

**Table 3:** Workbench test results for the two circular coils [30].

Circular Coil	Q	η (μT/W^1/2^)	$\eta /\sqrt{Q}$
Strip conductor	315	9.48	0.53
Wire conductor	345	11.01	0.57

**Table 4:** Workbench-measured coil efficiency [31].

Efficiency Measurement Method	η (μT/W^1/2^)
Perturbing loop	25.65 ± 1.76
Perturbing sphere	26.40 ± 0.52
Pickup loop	26.24
Nutation	25.10

**Table 5:** Efficiency measurements for the circular coil at two different z-coordinates [35].

Coil Efficiency	z = 0 cm	z = 1.3 cm
η (μT/W^1/2^)	30.13 ± 0.44	17.91 ± 0.63

## Data Availability

Not applicable.

## References

[1] Faghihi R., Zeinali-Rafsanjani B., Mosleh-Shirazi M.A., Saeedi-Moghadam M., Lotfi M., Jalli R., Iravani V. (2017). Magnetic Resonance Spectroscopy and its Clinical Applications: A Review. J. Med. Imaging Radiat. Sci..

[2] Jin J. (1999). Electromagnetic Analysis and Design in Magnetic Resonance Imaging.

[3] Haase A., Odoj F., Von Kienlin M., Warnking J., Fidler F., Weisser A., Nittka M., Rommel E., Lanz T., Kalusche B. (2000). NMR probeheads for in vivo applications. Conc. Magn. Reson..

[4] Roemer P.B., Edelstein W.A., Hayes C.E., Souza S.P., Mueller O.M. (1990). The NMR phased array. Magn. Reson. Med..

[5] Darrasse L., Kassab G. (1993). Quick measurement of NMR-coil sensitivity with a dual-loop probe. Rev. Sci. Instrum..

[6] Hoult D.I. (2000). The principle of reciprocity in signal strength calculations—A mathematical guide. Concepts Magn. Reson..

[7] Hoult D.I., Lauterbur P.C. (1979). The sensitivity of the zeugmatographic experiment involving human samples. J. Magn. Reson. (1969).

[8] Hornak J.P. (2002). Encyclopedia of Imaging Science and Technology.

[9] Maier L.C., Slater J.C. (1952). Field Strength Measurements in Resonant Cavities. J. Appl. Phys..

[10] Freed J.H., Leniart D.S., Hyde J.S. (1967). Theory of Saturation and Double Resonance Effects in ESR Spectra. III. rf Coherence and Line Shapes. J. Chem. Phys..

[11] Hornak J.P., Freed J.H. (1986). Spectral rotation in pulsed ESR spectroscopy. J. Magn. Reson. (1969).

[12] Alecci M., Seimenis I., McCallum S.J., Lurie D.J., Foster M.A. (1998). Nitroxide free radical clearance in the live rat monitored by radio-frequency CW-EPR and PEDRI. Phys. Med. Biol..

[13] He G., Evalappan S.P., Hirata H., Deng Y., Petryakov S., Kuppusamy P., Zweier J.L. (2002). Mapping of the B1 field distribution of a surface coil resonator using EPR imaging. Magn. Reson. Med..

[14] Hirata H., He G., Deng Y., Salikhov I., Petryakov S., Zweier J.L. (2008). A loop resonator for slice-selective in vivo EPR imaging in rats. J. Magn. Reson..

[15] Petryakov S.V., Schreiber W., Kmiec M.M., Williams B.B., Swartz H.M. (2016). Surface Dielectric Resonators for X-band EPR Spectroscopy. Radiat. Prot. Dosim..

[16] Sidabras J.W., Reijerse E.J., Lubitz W. (2017). Uniform Field Re-entrant Cylindrical TE[Formula: See text] Cavity for Pulse Electron Paramagnetic Resonance Spectroscopy at Q-band. Appl. Magn. Reson..

[17] Amida T., Nakaoka R., Komarov D.A., Yamamoto K., Inanami O., Matsumoto S., Hirata H. (2018). A 750-MHz Electronically Tunable Resonator Using Microstrip Line Couplers for Electron Paramagnetic Resonance Imaging of a Mouse Tumor-Bearing Leg. IEEE Trans. Biomed. Eng..

[18] Wiedemann H.T.A., Ruloff S., Richter R., Zollitsch C.W., Kay C.W.M. (2023). Towards high performance dielectric microwave resonators for X-band EPR spectroscopy. J. Magn. Reson..

[19] Chen C.N., Hoult D.I., Sank V.J. (1983). A further $\sqrt{2}$ improvement in sensitivity. J. Magn. Reson..

[20] Mispelter J., Lupu M., Briguet A. (2015). NMR Probeheads for Biophysical and Biomedical Experiments: Theoretical Principles & Practical Guidelines.

[21] Wright S.M., Wald L.L. (1997). Theory and application of array coils in MR spectroscopy. NMR Biomed..

[22] Nasserdine M., Menguè S., Bourcier C., Richalot E. (2014). Field Measurements within a Large Resonant Cavity Based on the Perturbation Theory. Progress. Electromagn. Res. B.

[23] Belevitch V. (1971). Lateral skin effect in a flat conductor. Philips. Tech. Rev..

[24] Doty F.D., Entzminger G., Hauck C.D., Staab J.P. (1999). Practical aspects of birdcage coils. J. Magn. Reson..

[25] Yeung D., Hutchison J.M.S., Lurie D.J. (1995). An efficient birdcage resonator at 2.5 MHz using a novel multilayer self-capacitance construction technique. Magn. Reson. Mater. Phys. Bioiogy Med..

[26] Giovannetti G., Francesconi R., Landini L., Santarelli M.F., Positano V., Viti V., Benassi A. (2004). Conductor geometry and capacitor quality for performance optimization of low-frequency birdcage coils. Concepts Magn. Reson. Part B Magn. Reson. Eng..

[27] Chen C.N., Hoult D.I. (1989). Biomedical Magnetic Resonance Technology.

[28] Giovannetti G., Frijia F., Menichetti L., Ardenkjaer-Larsen J.H., Hartwig V., De Marchi D., Positano V., Landini L., Lombardi M., Santarelli M.F. (2012). Coil Sensitivity Estimation with Perturbing Sphere Method: Application to 13C Birdcages. Appl. Magn. Reson..

[29] Hendrick E.R. (2008). Breast MRI: Fundamentals and Technical Aspects.

[30] Giovannetti G., Hartwig V., Landini L., Santarelli M.F. (2012). Classical and lateral skin effect contributions estimation in strip MR coils. Concepts Magn. Reson. Part B Magn. Reson. Eng..

[31] Giovannetti G., Frijia F., Hartwig V., Menichetti L., Ardenkjaer-Larsen J.H., De Marchi D., Positano V., Landini L., Lombardi M., Santarelli M.F. (2013). Efficiency evaluation of a 13C Magnetic Resonance birdcage coil: Theory and comparison of four methods. Measurement.

[32] Giovannetti G., Frijia F., Hartwig V., Menichetti L., De Marchi D., Positano V., Landini L., Lombardi M., Santarelli M.F., Ardenkjaer-Larsen J.H. (2012). A novel method for coil efficiency estimation: Validation with a 13C birdcage. Concepts Magn. Reson. Part B Magn. Reson. Eng..

[33] Keifer P.A. (1999). 90° pulse width calibrations: How to read a pulse width array. Concept. Magn. Res..

[34] Giovannetti G., Frijia F., Flori A., Montanaro D. (2019). Design and Simulation of a Helmholtz Coil for Magnetic Resonance Imaging and Spectroscopy Experiments with a 3T MR Clinical Scanner. Appl. Magn. Reson..

[35] Giovannetti G., Flori A., De Marchi D., Montanaro D., Frijia F. (2020). Design of a dedicated circular coil for Magnetic Resonance Spectroscopy studies in small phantoms and animal acquisition with a 3 Tesla Magnetic Resonance clinical scanner. Pol. J. Med. Phys. Eng..

[36] Frijia F., Flori A., Giovannetti G. (2021). Design, simulation, and test of surface and volume radio frequency coils for 13C magnetic resonance imaging and spectroscopy. Rev. Sci. Instrum..

[37] Giovannetti G., Flori A., Martini N., Cademartiri F., Aquaro G.D., Pingitore A., Frijia F. (2024). Hardware and Software Setup for Quantitative 23Na Magnetic Resonance Imaging at 3T: A Phantom Study. Sensors.

[38] Matson G.B., Vermathen P., Hill T.C. (1999). A practical double-tuned 1H/31P quadrature birdcage headcoil optimized for 31P operation. Magn. Reson. Med..

[39] Isaac G., Schnall M.D. (1990). A design for a double-tuned birdcage coil for use in an integrated MRI/MRS examination. J. Magn. Reson..

[40] Hoult D.I. (2000). Sensitivity and power deposition in a high-field imaging experiment. J. Magn. Reson. Imag..

[41] Kwok W.E. (2022). Basic Principles of and Practical Guide to Clinical MRI Radiofrequency Coils. RadioGraphics.

[42] Testagrossa B., Ruello E., Gurgone S., Denaro L., Sansotta C., Salmeri F.M., Acri G. (2021). Radio Frequency MRI coils and safety: How infrared thermography can support quality assurance. Egypt. J. Radiol. Nucl. Med..

